# Memory reactivation in slow wave sleep enhances relational learning in humans

**DOI:** 10.1038/s42003-024-05947-7

**Published:** 2024-03-08

**Authors:** Lorena Santamaria, Ibad Kashif, Niall McGinley, Penelope A. Lewis

**Affiliations:** Cardiff University Brain Research Imaging Centre (CUBRIC), School of Psychology, Cardiff University, Maindy Rd, Cardiff, CF24 4HQ UK

**Keywords:** Consolidation, Long-term memory

## Abstract

Sleep boosts the integration of memories, and can thus facilitate relational learning. This benefit may be due to memory reactivation during non-REM sleep. We set out to test this by explicitly cueing reactivation using a technique called targeted memory reactivation (TMR), in which sounds are paired with learned material in wake and then softly played during subsequent sleep, triggering reactivation of the associated memories. We specifically tested whether TMR in slow wave sleep leads to enhancements in inferential thinking in a transitive inference task. Because the Up-phase of the slow oscillation is more responsive to cues than the Down-phase, we also asked whether Up-phase stimulation is more beneficial for such integration. Our data show that TMR during the Up-Phase boosts the ability to make inferences, but only for the most distant inferential leaps. Up-phase stimulation was also associated with detectable memory reinstatement, whereas Down-phase stimulation led to below-chance performance the next morning. Detection of memory reinstatement after Up-state stimulation was negatively correlated with performance on the most difficult inferences the next morning. These findings demonstrate that cueing memory reactivation at specific time points in sleep can benefit difficult relational learning problems.

## Abstract (web summary)

Targeted memory reactivation (TMR) facilitates inferential thinking, and applying TMR on the up-phase of the slow oscillation leads to immediate benefit in this ability, whereas the same stimulation on the down-phase leads to an immediate detriment.

## Introduction

Relational memory is the ability to integrate multiple sources of knowledge, infer indirect associations between stimuli, and make decisions when presented with novel situations^1,2^. One example of such integration is transitive inference (TI), or the deduction of the rankings of non-adjacent members of a linear hierarchy which has been presented via exposure to adjacent pairs. In simpler words, knowing A > B and B > C can allow deduction that A > C in an A > B > C hierarchy. Despite being studied for many decades in humans^3^ and numerous other species^4–7^, the mechanisms for TI remain elusive^8,9^. Furthermore, a recent study demonstrated the dependence of TI on offline rest^7^, while a seminal study^10^ and its replication^11^, demonstrated that sleep is also beneficial to this task, however this is only true for the most distant inference pairs. While its role in TI remains unclear, sleep is thought to facilitate the abstraction of gist from recent experiences^12,13^, and the integration of these with prior knowledge^14,15^. Prior learning is spontaneously reactivated during sleep, which is important for such memory consolidation^16^. Notably, memory reactivation can be directly cued by re-administering sensory stimuli that have been paired with learned information using a technique known as Targeted Memory Reactivation (TMR), see^17^ for a meta-analysis.

Although some prior work has linked cognitive performance enhancement with rapid eye movement sleep (REM), e.g.^18–20^, most studies have focused on non-rapid eye movement sleep (NREM), and particularly on slow wave sleep (SWS), see^16,21–23^ for reviews. The main rhythms of SWS are slow oscillations (SO) and sleep-spindles. SOs are low-frequency oscillations at 0.5–1.5 Hz that reflect alternation between hyper-polarised neuronal down-phases and depolarised up-phases^24^. SOs drive transient sleep-spindles at 9–16Hz^25^ which have been associated with reactivation^16^. An elegant study^26^ recently showed that cueing in the SO up-phase elicited more spindles and less forgetting than cueing in the down-phase, strongly suggesting that up-phase cueing is more likely to facilitate memory consolidation.

Here, we set out to investigate how reactivation of a TI hierarchy during SWS, and particularly during SO up and down phases, influences the ability to make inferences. To this end, we adapted the experimental set-up from Ref. ^10^ to include three hierarchies so we could apply closed-loop TMR (CL-TMR) in a within-subject design. Thus, one hierarchy was stimulated during the up-phase (Up condition), another during the down-phase (Down condition), and the remaining hierarchy was not stimulated (Control condition). As recent work has shown that TMR effects can continue to unfold over time^27,28^, our participants performed a third behavioural test two weeks after the manipulation. In keeping with other studies^10,11^, we expected cued reactivation in sleep to benefit only the inference pairs. We also predicted a benefit in the Up condition but not in the Down condition, and we expected the benefits to last, or even increase, over time.

Our data show that TMR in Up is associated with classifiable memory reinstatement and better inferential reasoning, and that this is strongest in the most distant inference pairs. On the other hand, stimulation in Down produced no evidence of reactivation and led to a temporary inhibition of inferential reasoning that recovered after two weeks.

## Results

**Fig. 1:**
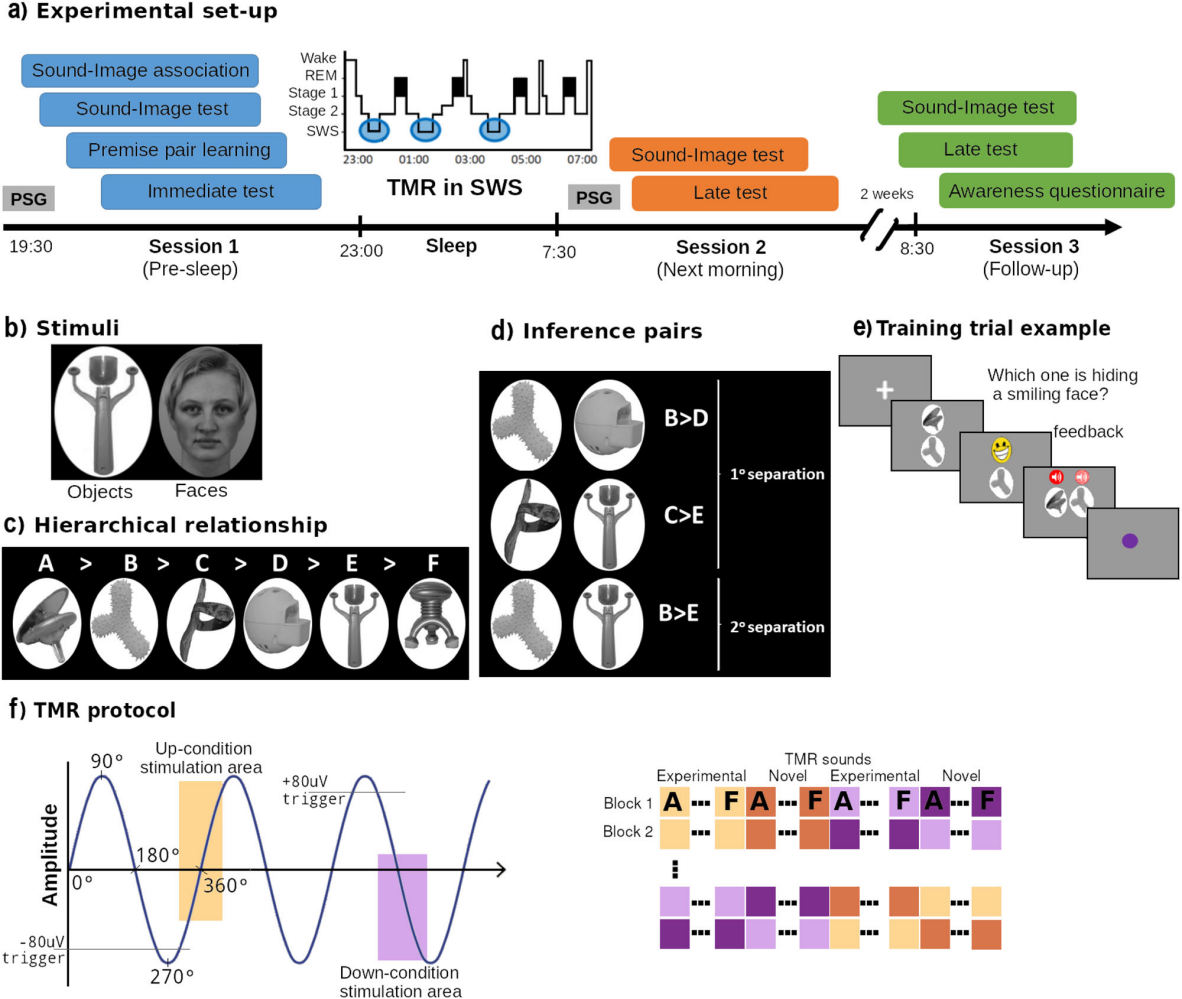
Experimental paradigm. **a** Timeline: first, participants were wired-up (EEG). Participants in the first session completed four behavioural tasks: sound-image association, sound-image test, premise pair learning and immediate test. During learning, premise pairs of three hierarchies were presented. The immediate test was the same as the learning counterpart but without feedback, allowing us to assess initial premise pair knowledge. Then, subjects went to sleep and TMR was carried out in SWS, where two out of the three hierarchies were stimulated. After waking up, still with the electrodes on, participants completed Session 2, which comprised a sound-image association test and a Late test. During the Late test, Inference pairs were presented for the first time, together with the previously learned premise pairs. After 2 weeks they returned to the lab for a third session, without EEG recording, where they completed the same tests as in Session 2 plus the awareness questionnaire. **b** Example of two of the categories used in the experiment: uncommon objects^49^ and female faces^48^. **c** Example of the visual stimuli arranged in a randomly determined hierarchical order. **d** Resultant inference pairs from (**c**). **e** Example of a typical learning trial. A cross appearing on the screen indicates the start of the trial (lasting between 0.5 s to 1 s), then two images of the hierarchy appear on the screen lasting until the user presses the up or down arrows. Feedback is provided immediately afterwards (a happy or angry face accordingly). To help sound-image consolidation, the sound of each stimulus is played while the stimulus is presented again on the screen. Finally, a purple dot is presented for 500 ms to indicate the end of the trial. During the test part, the feedback and the sounds are eliminated. **f** TMR protocol: left side showing the SO detection system used for the up (yellow) and down (purple) transition areas. On the right side is an example of TMR blocks, for block 1 first were played the 6 items for the Up experimental condition, then Up-novel sounds, followed by the Experimental and Novel sounds of the Down condition, respectively. Each element of the hierarchy was played in the right order for the experimental condition (A, B, ...F), additional novel sounds were assigned to each hierarchy. The images used to create the section B: the female face is the element AF28NES from The Karolinska directed emotional faces (KDEF)^48^, the rare object is from the Novel Object and Unusual Name (NOUN)^49^. The images from sections D and E were again taken from the NOUN database.

Prior to sleep, participants performed a transitive inference task (Fig. 1a) with three hierarchies (Fig. 1b) of 6-items each (Fig. 1c). Each of the items was associated to a separate semantically related sound (e.g., traffic noise with a city landscape). Participants learned the sound-image associations until they achieved over 90% accurate in a retrieval task. They then learned the transitive inference task by repeatedly viewing adjacent (premise) pairs (e.g., A-B) for each hierarchy, and indicating which hid the smiley face, with feedback (Fig. 1e). After a 5-minute delay, the same task was performed without feedback to assess learning level (immediate test). The next morning, participants performed this test again, but inference pairs were now included: B-D, C-E and B-E (late test, Fig. 1d). Sounds associated with two out of the three hierarchies were presented during SWS, with one hierarchy presented in Up and another in Down using CL-TMR. To control for other EEG underlying mechanisms induced by the cueing, novel sounds were also played during the night for both Up and Down states.

### Behavioural results Premise pairs

Immediate test performance on premise pairs was above chance (50%) in all three conditions (Down: 74.7 ± 0.02%, Control: 79.2 ± 0.02%, Up: 79.6 ± 0.02%), but not at ceiling (see Table 2-2). A 1-way ANOVA showed no significant difference between these conditions at baseline (*F* = 2.69, *p* = 0.07), a post-hoc analysis (2-tailed t-test) corroborated that there were no significant differences: Down vs Control (*p* = 0.095), Down vs Up (*p* = 0.095), Up vs Control (*p* = 0.93). Hence, participants had equal premise pair knowledge for all conditions in the immediate test. Performance on the sound-image association was over 90% for all the stimuli (Supplementary Note 2, Supplementary Fig. 1) and the number of times each stimulus was used for each condition remained relatively constant, Supplementary Table 1. Hence, we can rule out any bias due to stimuli or cue-memory associations.

**Fig. 2:**
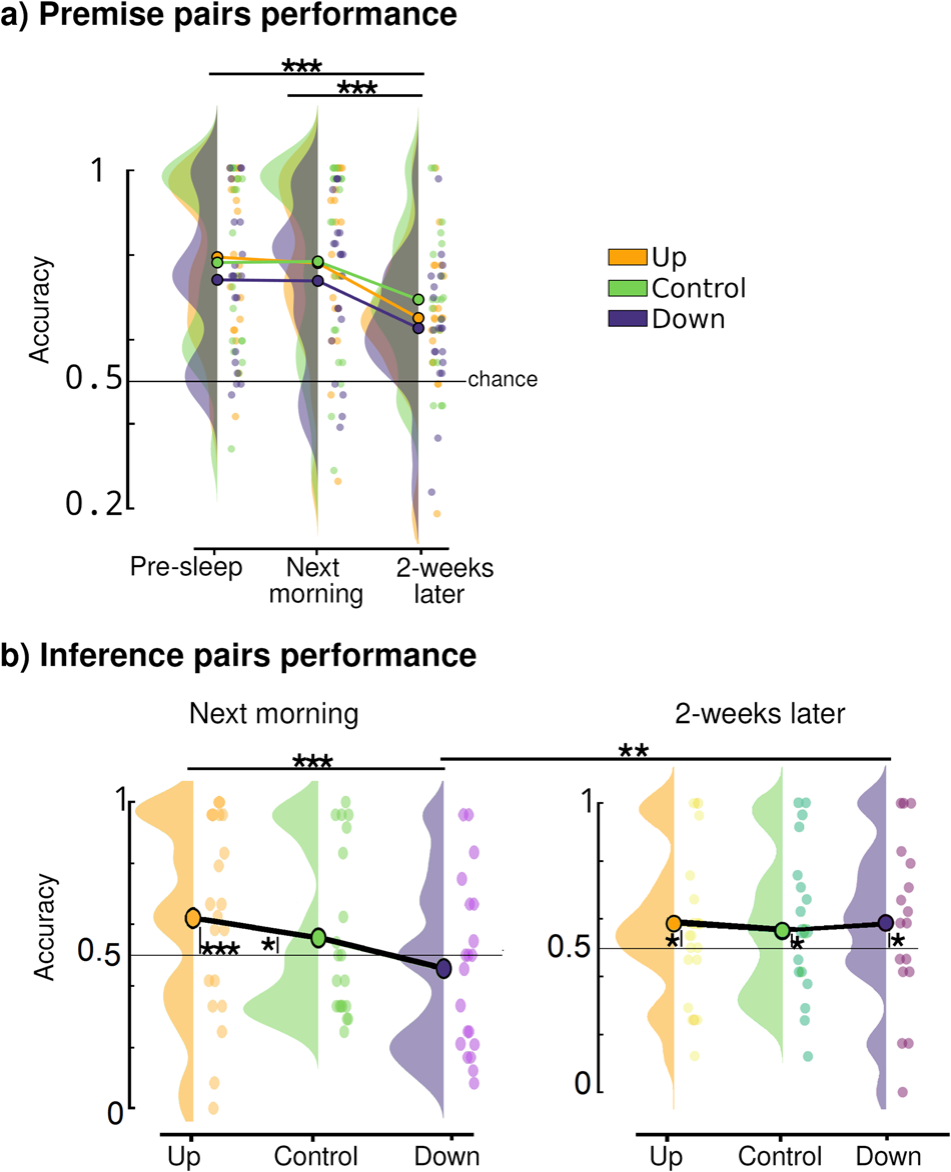
Behavioural performance. **a** Premise pair accuracy results for each Session (Pre-sleep, next morning and two-weeks later) and for each Condition (Up in yellow, Control in green and Down in purple). **b** Inference pair performance for Session 2 (Next morning, left) and Session 3 (2-weeks later, right) for each Condition. Statistically significant differences are indicated as *p < 0.05, **p < 0.01 and ***p < 0.001. In (**b**) additional statistical significance from chance level (50%) were shown per each condition.

To examine how in the premise pair performance evolved over time, and whether there were any impacts of TMR on this, we performed a repeated measures (RM)-ANOVA with Session (3 levels) and Condition (3 levels) as within subject factors, and with accuracy as the dependent variable (see Fig. 2a, Supplementary Note 3). Note that 20 participants completed Sessions 1 and 2, while only 17 completed Session 3 as well. We found main effects of Condition (*F*(2,435.89) = 3.5, *p* = 0.029) and Session (*F*(2,556.98) = 32.39, *p* < 0.0001), but no interaction (*F*(4,432.5291) = 0.42, *p* = 0.72). Further analysis for Condition revealed no significant differences between conditions in any of the three sessions (smallest *p* = 0.103). On the other hand, there were clear Session effects: both between Session 1 (pre-sleep) and Session 3 (two-weeks later), and between Session 2 (next morning) and Session 3, Fig. 2a (all *p* < 0.001). Thus, in keeping with normal declarative forgetting, there was a marked drop in premise pair performance over two weeks irrespective of condition, see Supplementary Table 2 for details. There was also no overnight improvement in premise pair accuracy, but this is in line with previous TI literature^10,11^. This result might be surprising given that associative memories are often strengthened by sleep^16^, but is also in line with the idea that sleep facilitates more weakly encoded memories^29^, though this could also depend on other factors such as the type of task or the strength of the cue-memory associations^30^.

### Behavioural results Inference pairs

**Fig. 3:**
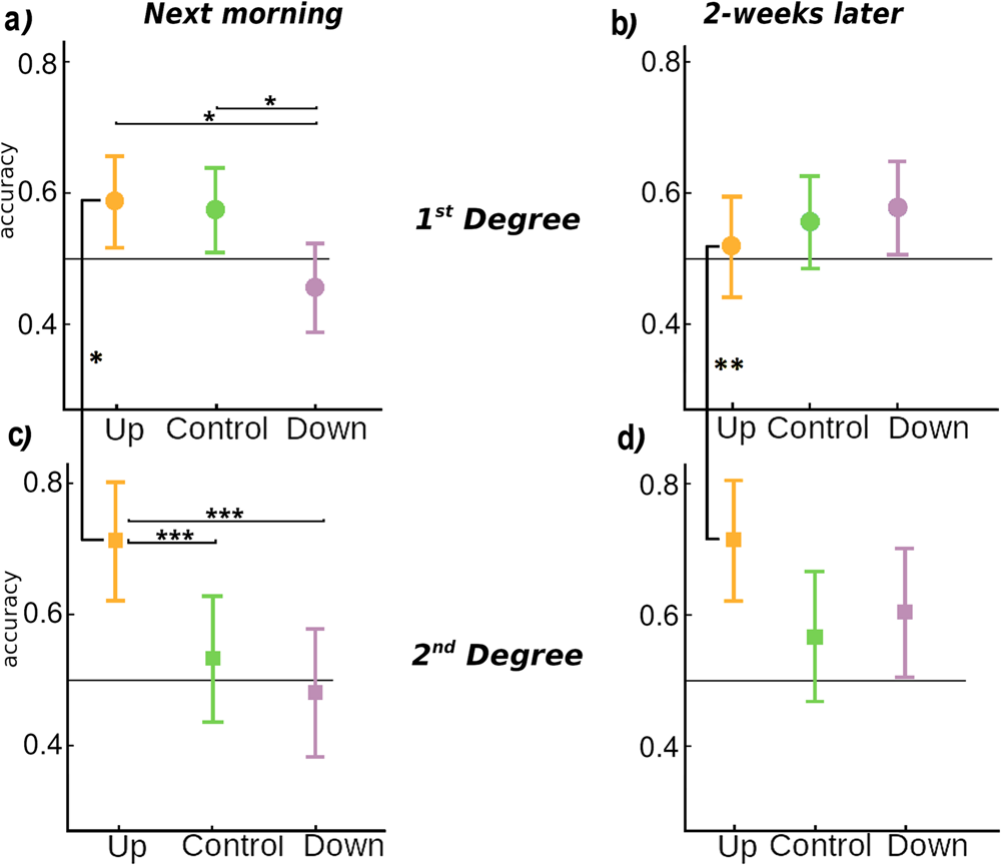
Behavioural results. Inference pair performance for each condition: Up (yellow), Down (purple) and Control (green). Inference pairs performance separated by degree of separation: **a** 1^st^ degree of separation for Session 2 (next morning) and (**b**) Session 3 (follow-up), **c** 2^nd^ degree of separation for Session 2 and **d**) Session 3. Bars represents 95% confidence intervals. Statistically significant differences are indicated as: *p < 0.05, **p < 0.01 and ***p < 0.001. All results are corrected for multiple comparisons. An extended version of the figure can be found in Supplementary Fig. 2.

Inference pairs were introduced in the Late test performed during Session 2 and repeated in the 2-week follow up (Session 3). Results are shown in Fig. 3 and Supplementary Note 4. To examine the effect of TMR on inference pair performance both the next day and two weeks later, and how this differed for close (1st degree, Fig. 3a, b, Supplementary Fig. 2a, b) and distant (2nd degree, Fig. 3c, d, Supplementary Fig. 3c, d) inferences, we performed a RM-ANOVA with the factors Session (2 levels), Condition (3 levels) and Degree of separation (2 levels) (see Fig. 1d, Supplementary Tables 3 and 4). This revealed main effects of Degree (*F*(1,560.27) = 5.804, *p* = 0.016) and Condition (*F*(2,460.13) = 13.97, *p* = 0.002) but not Session (*F*(1,635.97) = 1.96, *p* = 0.17). This ANOVA also revealed two interactions: between Degree and Condition (*F*(2,460.13) = 9.89, *p* = 0.008), and between Session and Condition (*F*(2,437.06) = 7.86, *p* = 0.021), but no interaction between Session and Degree (*p* = 0.601). Finally, the interaction between all three factors was not significant (*p* = 0.722). We examine the significant results in further detail below.

To directly investigate the interaction between Degree and Condition, our post-hoc t-tests collapsed across Session. This showed that the Up condition differed from both Control (*F*(1,144) = −3.27, *p* = 0.014) and Down (*F*(1,144) = −3.27, *p* = 0.017) at the 2nd Degree only. There was also a difference between 1st and 2nd degree in the Up condition (*F*(1,323.87) = −3.624, *p* = 0.004), but there were no differences between the Control and Down conditions for either Degree of separation (lowest *p* = 0.79). All probabilities were corrected for multiple comparisons, see Figs. 2b and 3 and Supplementary Tables 5 and 6. These results clearly demonstrate that Up-phase TMR can provide a benefit to transitive inference, but in keeping with^10^, this is only significant for the most distant items.

To examine how the effects of Condition on 2^nd^ degree items change over time, we performed separate ANOVAs for 2^nd^ degree items at Sessions 2 and 3. At Session 2, this showed better performance for Up vs. Control (F(1,77) = −0.416, *p* = 0.027) and Down (F(1,77) = −0.494, *p* = 0.006), but not between Down and Control (F(1,77) = 0.077, *p* = 0.880), all corrected for multiple comparisons, Fig. 3c, Supplementary Fig. 2c and Supplementary Table 7. At Session 3, two-weeks later, comparisons of Up vs. Control and Up vs. Down were no longer significant (F(1,67) = −2.14, *p* = 0.087 and F(1,67) = −1.59, *p* = 0.251, respectively). The benefit of the Up stimulation therefore fades over the two-week consolidation period (Fig. 3d, Supplementary Fig. 2d).

In summary, stimulation of the SO up state was associated with significantly better performance on the most distant (2nd Degree) inference pairs compared to Control stimulation in the next-day data, Fig. 2b lower left panel. Interestingly, stimulation of the Down phase appeared to impair inferences when compared to Up, irrespective of degree of separation (Fig. 2, left panel). However, this picture changed after two-weeks, when performance on inference pairs stimulated in the Down phase improved to above chance levels and did not differ from performance on pairs in the Control and Up conditions (Fig. 2a, right panel). Performance on Up and Control conditions did not change over this retention interval.

Interaction between Session and Condition; post-hoc analysis of the interaction between Session and Condition revealed a difference between Up and Down conditions (*p* < 0.001, corrected for multiple comparisons) at Session 2, supporting the idea that the Up and Down states are associated with distinct forms of neural processing^26^ (see Fig. 2b). However, Up-Control and Down-Control showed not statistically significant differences (*p* = 0.46 and *p* = 0.19, respectively). No differences were found for Session 3. This analysis also revealed an unexpected difference between Sessions for the Down condition (*F*(1,430.25) = −3.012, *p* = 0.038), with a significant improvement of performance from under chance level (50%) at Session 2 (46.4 ± 2.8%) to over chance level two-weeks later (58.6 ± 2.9%). Control and Up conditions remained over chance level across both sessions, Figs. 2b and 3a and Supplementary Tables 5–7 for full report.

Finally, we investigated a global TMR effect. Because the 1st Degree items also showed a trend towards benefit from TMR, we collapsed across both Session and Degree for an exploratory analysis to test our a-priori hypothesis that Up state cueing would benefit all inferences. This showed better performance after Up than Control across all stimuli (*F*(1,436) = −2.56, *p* = 0.03). Performance was also better for Up vs Down stimulation (*F*(1,436) = −3.388, *p* = 0.002), but not for Control vs Down (*F*(1,436) = 0.826, *p* = 0.687), all corrected for multiple comparisons.

### EEG results

To distinguish the effect of TMR upon EEG responses from the effect of a playing a sound, we used both Experimental sounds, which were associated with the previously learned hierarchy, and Novel sounds, which were associated with an unlearned hierarchy as indicated in Fig. 1f.

**Fig. 4:**
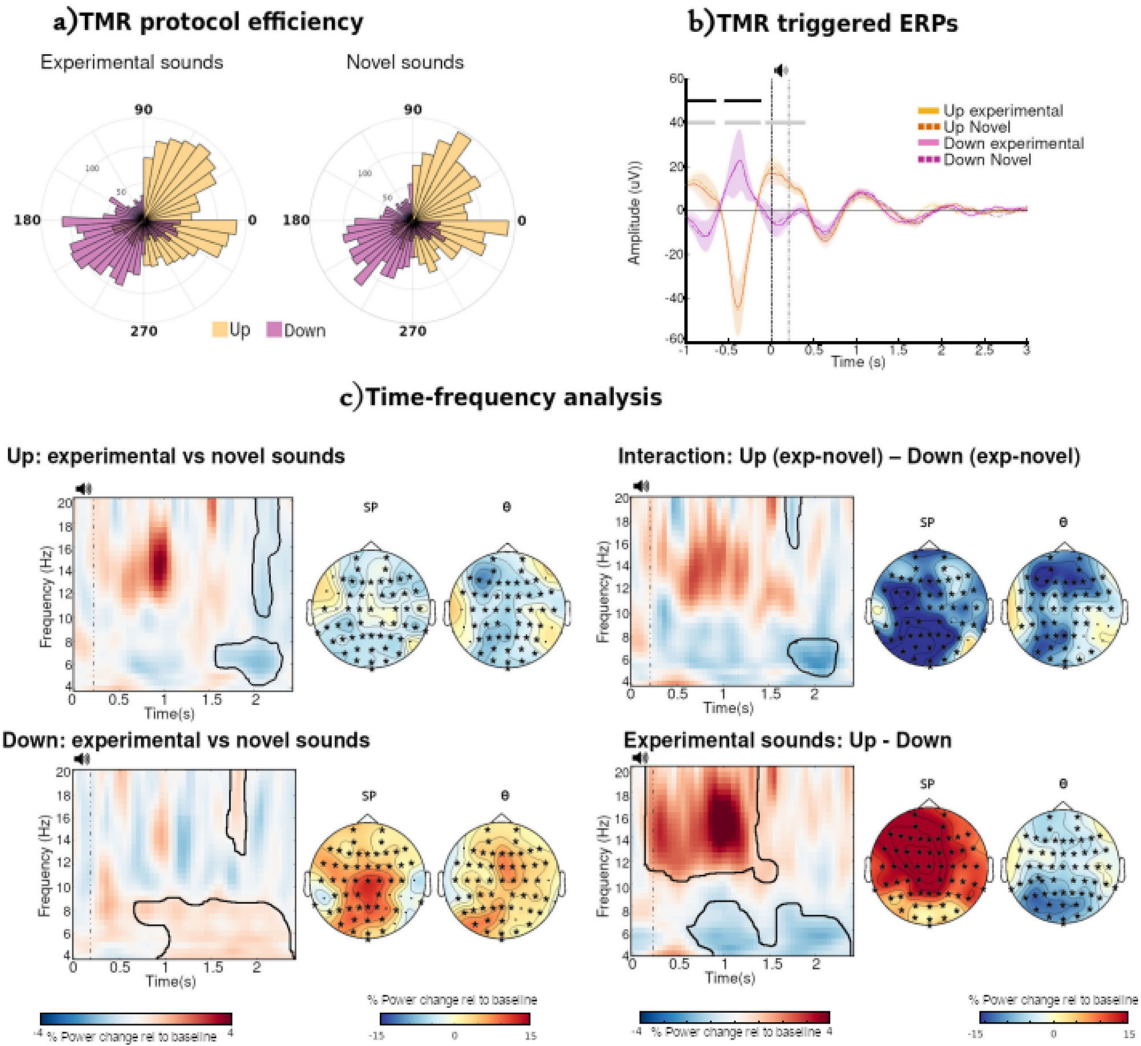
EEG analysis. **a** Phase angle analysis at stimulus release per each trial. The left polar histogram represents the Experimental sounds with Up condition in yellow and Down condition in purple. Similarly, the right histogram represents the Novel sounds. **b** ERP statistics. ERPs for Up (yellow) and Down (purple) Conditions and Experimental (solid line) and Novel (dashed line) sounds. All ERP-data are shown as mean ± SEM (standard error of the mean) across participants. There are no differences between Experimental and Novel sounds but there are differences, as expected, between Up and Down stimulations. These differences are highlighted in grey for the Novel sounds and black for the Experimental sounds. All graphs are the values for electrode F3. **c** Time-frequency analysis results (grand average at F3 channel). Experimental vs. Novel sounds power spectrum differences are plotted for Up condition, Down, their interaction and a direct comparison Up vs Down for Experimental sounds. Vertical dashes lines indicate the onset of the auditory TMR cue (200 ms). The black contour outlines significant clusters (two tailed, *p* < 0.05). The power spectrum differences from those significant clusters were used to plot the topographies on the right side of each graph. Topographies were divided into theta (θ:5–8 Hz) and a general spindles frequency band (SP:10-20 Hz). Channels involved in the significant cluster are highlighted with an asterisk.

To check that our online algorithm correctly differentiated between the Up and Down conditions, we calculated event-related potentials (ERPs) separately for each Condition (Up/Down) and Cue type (Experimental/Novel sounds) (Fig. 4b). In addition to the expected ERP differences between Up and Down before cue onset, there was a third significant cluster for Novel but not Experimental sounds. Furthermore, after the sound offset, both cues and type of sounds elicited a second SO cycle that made them statistically indistinguishable in line with previous literature^26,31^. To corroborate the accuracy of our closed-loop algorithm, we calculated the phase of the cortical SO at stimulus onset for each trial and participant (Fig. 4a). For Experimental sounds, the average values at channel F3 were 358.20° (standard deviation (SD):0.58) and 205.61° (SD:0.44) for the Up and Down conditions respectively. Similar values were obtained for the Novel sounds: 358.79° (SD:0.59) and 208.37° (SD:0.50) respectively. Circular statistics corroborate a significant difference between Up and Down conditions for both Experimental and Novel (*p* < 0.001) but no differences between Experimental and Novel in either Down or Up condition (*p* > 0.1).

Time-frequency analysis of the Experimental vs. Novel sounds was performed for frequencies between 4 and 20 Hz for Up and Down cueing (see Fig. 4c and Supplementary Fig. 3) for all available channels. For Up, there was a significant power decrease in both SP (cluster *p* = 0.008, 2.05 s to 2.1 s) and theta bands (cluster *p* = 0.008, 1.6 s to 2.3 s) towards the end of the trial, Fig. 4c. For Down, the opposite was observed, with a power decrease apparent (*p* = 0.012, *t* = 1.7 s to 1.8 s and *p* = 0.010, *t* = 0.7 s to 2.35 s). There also was a significant interaction between cueing Condition and type of sound in theta band (*p* = 0.007, *t* = 1.6 s to 2.3 s), but not significant interaction between these in spindle band (*p* = 0.077, *t* = 1.7 s to 1.8 s). These temporal late differences are in line with previous work^31,32^. We also compared Up and Down directly for the Experimental sounds to assess the beneficial effect of the Up condition. There is a positive cluster (Experimental > Novel) around the sound onset (*p* = 0.008) for SP band lasting until around 1.25 s. This is similar to other CL-TMR literature^26^. On the other hand, for theta band there is a decrease in power (Experimental < Novel) with a significant cluster (*p* = 0.037) ranging from 0.65 s to 2.4 s.

**Fig. 5:**
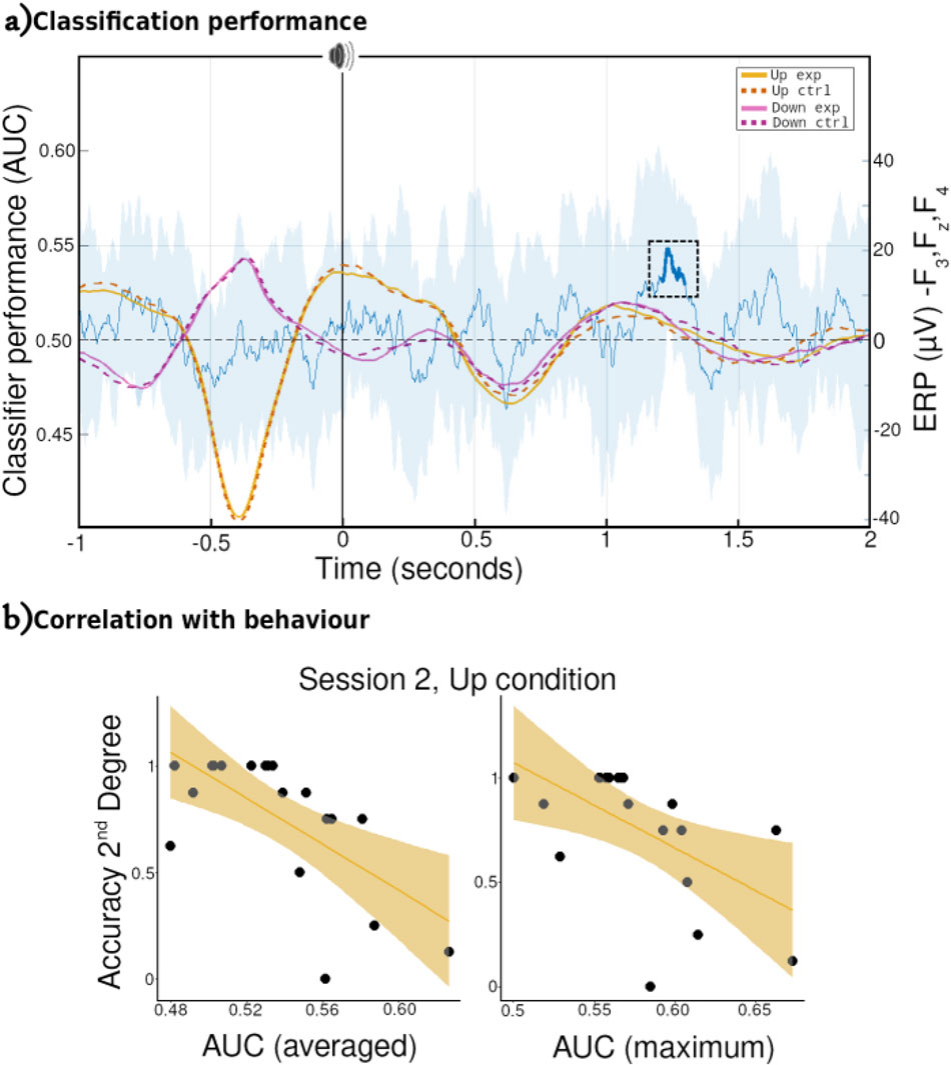
Classifiers. **a** Up-Down classification performance. SVM classifier (blue line) presented a statistically significant cluster (over chance level) centred around 1.3 s (thicker blue line). On top are superimposed the grand average ERPs value (mean of F3, Fz and F4 channels) for Up (yellowish colours) and Down (purple colours), with solid lines representing experimental sounds and dotted lines novel sounds. **b** Correlations between the SVM (AUC) mean and peak performance, respectively, of the significant cluster for the Up condition with the behavioural accuracy of the second degree of difference-inference pair of the Up condition. Shadow areas represent 95% confident intervals.

To determine if memory-related neural activity was reactivated during the night, we trained two machine learning algorithms to differentiate between Experimental and Novel sounds for each condition (Up/Down). After calculating the classifiability of data for each participant, we performed cluster statistics at the group level for each condition^33^. Only the Up condition presented a significant cluster of above-chance classification. This cluster (*p* = 0.037) ranges from 1204 ms to 1298 ms after stimulus onset for the SVM classifier with area under the curve (AUC) as performance metric (Fig. 5a). Similar values were obtained for the rest of the tested combinations, Supplementary Tables 8 and 9 & Supplementary Fig. 4 from Supplementary Note 5. There were no significant clusters for the Down condition (lowest *p* = 0.074). The absence of significant classification here could indicate that the brain is not able to activate the corresponding memory trace above chance when the cues are presented in the down phase. Hence, no behavioural benefit is obtained the following morning. However, this does not explain the performance improvement observed two weeks later.

To better understand the relationship between reactivation and consolidation, we performed a series of correlations between classification performance within the significant cluster (mean and peak) and the behavioural metrics described above. This revealed a negative correlation (*R* = −0.65, *p*_corrected_ = 0.022) between mean classification (SVM + AUC combination) and behavioural performance on 2^nd^ degree inference pairs in Session 2, Fig. 5b. This fits well with the fact that only 2^nd^ degree pairs in the Up condition presented a significant difference in overnight improvement when compared with the control condition for Session 2, and similarly only Up showed between condition differences in the degree of separation of inference pairs. The correlation was constant across all tested machine learning algorithms, see Supplementary Tables 10 and 11. No other correlation with any behavioural metric was significant (all *p* > 0.05 before correction). The fact that this correlation is negative, e.g., the better the algorithm can classify the worse participants performed on that particular pair, is perhaps surprising. Similar negative correlations have been reported, e.g., between classification performance and behavioural metrics^34^. One possible hypothesis is good performers try to fit the Novel sound into the previously learned hierarchy. That is, when they hear the Novel sound, they may reactivate the cued hierarchy again, making it more difficult for the classifiers to differentiate between control from learned sounds.

## Discussion

Transitive inference is a key cognitive ability and a hallmark of deductive reasoning^35^. By showing that TMR in SWS strengthens such inferential thinking, our data support the idea that memory reactivation is important for more than the mere strengthening of memories which has been investigated by the bulk of the TMR literature^17^. It is also involved in more complex integration and restructuring processes^36^. Furthermore, our findings show that such integration can be intentionally boosted through an external intervention, an observation which may be important for the development of cognitive enhancers targeting this kind of thinking. The fact that our manipulation had a stronger effect on the more distant 2^nd^ degree inferences is exciting in that it suggests the power of reactivation to assist with the integration of quite distant pieces of information.

Phase of the slow oscillations and memory: the concept of an optimal phase or window for TMR stimulation stems from the fact that depolarising Up-states are more likely to activate larger groups of neurons synchronously^37^ and to drive thalamocortical spindles and sharp-wave ripples in the hippocampus^38^, thus facilitating hippocampal memory reactivation. This could explain why our classifiers only detect memory reactivation after Up stimulation, and why we found next morning behavioural benefit only after stimulation in this phase. Notably, our ANOVA showed a trend towards worse performance on premise pairs related to the Down hierarchy compared with Up and Control hierarchies at baseline. It is possible that worse memory at this timepoint might lead to worse subsequent inference performance for the Down condition. However, when we tested for a correlation between premise performance at baseline and subsequent inference performance there was no significant relationship for either Up or Down at either Session 2 or Session 3 (smallest *p* = 0.187), making this explanation unlikely. All in all, our findings are in keeping with recent work showing that Up phase stimulation elicits classifiable reactivation and boosts memory performance, while Down phase stimulation does not^26^ as well as work showing that Up phase TMR more effectively promotes emotional updating than stimulation at other SO phases^39^. Thus, our results join a growing literature showing that Up phase TMR has a stronger impact on neural processing.

By contrast, down-phase stimulation appears to lead to a temporary impairment in performance, which is interesting in its own right. Notably, however, both the benefit of up-state stimulation and the detriment of down-state stimulation disappear after two weeks (see below).

The question of how long sleep related impacts on memory lasts is increasingly topical^40,41^. One study reported that a TMR related reduction of implicit bias was retained after a week^17^, while another study reported that TMR benefit to a serial reaction time task peaked after ten days^27^, and a more recent paper from our group showed that TMR benefit to gist abstraction actually strengthened across a 1 week delay^42^. Here, accuracy for inference pairs was maintained two weeks after the manipulation for both Up and Control conditions (Figs. 2 and 3). However, performance in Down increased from below chance the morning after stimulation to above chance two weeks later, reaching similar overall accuracy to the other two conditions (Fig. 3a, Supplementary Fig. 2a). This improvement suggests that despite an initial inhibition due to TMR in the Down phase, the neural representation was able to recover over time. It is possible that subsequent nights of sleep without any manipulation may have allowed spontaneous reactivation of the memories, and that this occurred equally for all conditions. While Up and Control conditions, which had reactivated successfully in the first night, derived no benefit from this additional reactivation, the Down condition did benefit, and the associated consolidation allowed the Down-stimulated hierarchy to essentially catch up with the other hierarchies. Additionally, the next morning test in Session 2, which included presentation of inference pairs, may also have helped to trigger subsequent reactivation, from which Down condition benefited.

The time-frequency response to our stimulation differed markedly between Up and Down stimulations. Up stimulation was associated with a significant transient decrease in both Theta and Sigma activity around 2 seconds after the TMR cue, while Down stimulation led to a long significant increase in theta ~1–2.5 s post-cue, and a much more transient increase in Sigma around 1.6 s post-cue. It is difficult to compare these findings directly with the literature since only two prior studies have applied closed-loop TMR and included a time-frequency analysis, and these used quite different tasks and methods from ours. Goldi et al.^43^ averaged across all electrodes, Ngo et al.^44^ showed results only for electrode Cz, and we presented results from F3. Furthermore, Goldi et al.^43^ used a word pair learning task and compared the pattern associated with subsequently remembered vs non-remembered words before comparing the result of this across Up and Down stimulation phases. Ngo et al.^44^ used image-word associations and presented the data associated with Up and Down stimulation separately, then in comparison. Our study set out to examine memory related responses to stimulation, and we therefore included Novel control sounds that were stimulated at Up and Down phases so that we could subtract these from the responses to Experimental Up and Down stimulations that were paired with memories. However, we also computed the simpler subtraction of Up stimulation vs. Down stimulation that was chosen by Ngo et al.^44^, Fig. 4c bottom right panel. When we focus on this subtraction, our results do not look so different from those of the prior studies^26,31^, even despite all the methodological differences. Thus, we find an increase in sigma and a decrease in theta after Up (compared to Down) stimulation in all three studies. This supports the idea that the phase at which TMR is applied plays an important role in determining the impact it will have on the brain, at least in the short term. It is noteworthy that neither our data nor that of other closed-loop TMR studies shows the same pattern of EEG results as open-loop stimulation^32,45^.

The study has a series of limitations, for instance by task design the number of the 2^nd^ Degree instances in half than for the 1^st^ Degree, which may have introduced noise into the data. Another caveat that was not controlled for was the menstrual phase for the female participants, which has been reported as a mediator of TMR effectiveness^46^. In order to mitigate individual variability and other possible external factors than can affect the data we specifically designed a within-subjects experiment. However, it will be interesting to see in future experiments if changing the type stimuli or controlling for menstrual phase introduce any variation on the results.

Our results show that the complex process of making indirect inferences can be facilitated by cued reactivation during slow wave sleep. Importantly, this was only true for the most distant inference pairs, and Up phase cueing was effective immediately (next morning) while Down phase cueing caused an initial impairment. Two weeks after stimulation all Up cueing benefits had vanished but more interestingly the Down state condition improved to over chance level, though not above the level of uncued items. These results show that TMR effects evolve over time, and further studies will be needed to understand them. These results provide strong support for the idea that memory reactivation in sleep is important for high-level qualitative changes in memory, such as integration and relational memory. Our findings also hold promise for the use of sleep-based interventions to drive improvement in such complex memory and its application to real-word tasks.

## Methods

### Participants

Thirty adults (10 males, mean age 27 ± 3.72) participated in the overnight experiment. All had no self-reported history of neurological, sleep or motor disorders. All participants completed a screen questionnaire before selection, provided written informed consent, and were reimbursed for their time. The experiment was approved by the School of Psychology Ethics Committee at Cardiff University. Participants lived within easy travelling distance of the University and agreed to abstain from caffeine and alcohol during the study and for 24-hours before. From the thirty participants that completed the task, 10 were eliminated either because of technical problems (*n* = 3) or because they did not have enough stable SWS (*n* = 7) to perform the stimulations (we required 12 rounds, these participants were mostly in light sleep and N2 stage). From the 20 participants left, 3 could not finish Session 3 due to the pandemic. Thus 20 participants completed Sessions 1 and 2, and 17 completed Session 3 as well. We performed a post-hoc analysis in R to calculate the minimum detectable effect size given power (0.8), sample size (20), groups (3) and sessions (3). For a repeated-measures ANOVA analysis this gave *f* = 0.91.

### Materials

The behavioural tasks were presented in a quiet room, participants were comfortably seated in front of the computer and stimuli were presented using Matlab^©^, Psychtoolbox^46^ and Cogent 2000 (www.vislab.ucl.ac.uk). Three types of visual stimuli were presented to the participants: female faces^47^, outdoor scenes (taken from the internet) and unusual objects^48^, see Fig. 1b. Each stimulus was easily distinguishable from others within and between categories. All items were presented in greyscale and matched for luminance. Each image was associated with an exclusive sound, semantically congruent with the image as closely as possible (e.g. bike image with a bike bell sound).

Sounds were taken from the internet and truncated into two different lengths, 2 s and 200 ms, and pitch normalised. We used the longer sounds in behavioural training, facilitating the sound-image encoding, and the shorter version for the rest of the behavioural tasks and TMR cueing. The sounds were played through noise-cancelling headphones (Sony MDR-ZX110NA) during behavioural tasks and through speakers (Dell A225) during sleep. The order of presentation of each stimulus category was counterbalanced across participants and the order of stimuli within each category was completely randomised for each subject. Hence, the experimenter was completely blind to which stimuli form the hierarchy and its order within each condition (Up, Down, Control), and which type of stimuli (faces, objects or scenes) was selected for each condition, so as not to influence the results. Each of the three hierarchies (Fig. 1b, c) comprised 6 images, each one with an associated (highly discriminable) sound. We prepared a set of 12 images and 12 sounds per hierarchy, which is a total of 36 images and sounds. At the beginning of the experiment, for each one of the three hierarchies, 6 of these images with their corresponding sounds were selected to be learned and the remaining 6 sounds were used as controls to be played during the TMR stimulation. Before participants started the first task, the 6 images which would be used during the experiment and the other 6 which would be used as control were randomly selected for each category (the experimenter was blind).

### Procedure

Participants arrived at the laboratory around 8 pm and changed into their sleepwear. They reported alertness by completing the KSS^49^ and SSS^50^ questionnaires. Afterwards, they were fitted for PSG recording and performed both the initial training and the immediate test explained in Experimental tasks section and Fig. 1a. Participants were ready for bed around 11 pm. During the night, the previously learned tones were played softly during SWS. From the three stimulus categories, one was kept as a control (Control) and was not played during the night, allowing us to compare it against the other two which were cued during the Up and Down phases of the SOs respectively. After 7–8 hours of sleep, participants were woken at the agreed time and allowed > 20 min to overcome sleep inertia. During this time, they could go to the toilet, eat, and drink before completing the sleep quality, KSS and SSS questionnaires. Participants then completed the Late test and another Sound-Image association test (Experimental tasks section and Fig. 1a). Afterwards, the electrodes were removed, and participants could shower and go home. Finally, participants came back to the laboratory two weeks later ( ± 2 weeks) to complete the second Late test and Sound-Image association test, identical to the previous one but without EEG recordings, to test the robustness of sleep-TMR mediated benefits.

### Experimental tasks by session

The experiment was composed of three sessions: evening (Session 1), next morning (Session 2) and a follow up session two-weeks after (Session 3). Each of the sessions was divided into different tasks as below:

Session 1- Sound-Image association learning task: For each of the three categories, participants were shown each of the six items forming the category one by one. At the same time the associated sound (2 s length) was played. Each Sound-Image pair was shown 4 times. The order of items within a category was randomized and the order of the categories themselves was counterbalanced across participants.

Session 1- Sound-Image association test: Immediately after training, all participants performed a recall session to determine retention level. Three images were presented on the screen while a sound was played. Participants were asked to select as quickly and accurately as possible the image corresponding to the sound using the keyboard arrow keys. When they responded, a rectangle surrounded the correct image (green if the participant’s selection was correct or red if it was wrong). Image screen position was randomized on every trial. The three images presented on the screen were pseudo-randomly selected, with the restriction that at least one of the two images was a lure of the same category as the right answer. The sounds were cut down to only 200 ms long. Participants performed two blocks with three repetitions of each sound per block. At the end of each block, accuracy was presented.

Session 1 – Premise pair learning task: Following previous related experiments^10,11^, all participants learned five relational premise pairs for each of the three categories. If each category formed a 6-item hierarchy, schematically represented as A > B > C > D > E > F (see Fig. 1c), the premise pairs would be: A > B, B > C, C > D, D > E, E > F where the notation ‘A > B’ indicates ‘select A over B’. The pairs were presented one at a time, with images stacked vertically (Fig. 1e). Subjects were instructed to select the item hiding a smiley emoticon from the two presented, at first by trial and error, but after practice and feedback they learned which item was correct. If they selected the correct item, it was replaced by a smiley emoticon. This is in line with^11^, where a smiling-emoticon was used as reinforcement. If they selected the wrong image it was replaced by an angry emoticon. After the feedback, participants received a second reinforcement as the pair was presented again but this time horizontally instead of vertically, and in the correct order (e.g. A-B) from left to right, with the corresponding sounds also played in the correct order. Pairs were organised into blocks of 10 trials for each of the hierarchies. This meant a total of 30 trials per block. Each block presented each of the five pairs of each hierarchy twice, counterbalancing the up-down positions (e.g., A above B and B above A, with A being the correct selection in both cases). The three hierarchies were not mixed within a block. For example, first all pairs for the scenes category were presented, then pairs in the faces category, and finally the object pairs. This order was counterbalanced across participants. Within each category, pairs were ordered pseudo-randomly to avoid explicitly revealing the hierarchy. Hence, a displayed pair cannot contain an item that was in the previous pair (e.g., A > B will never be followed by B > C). Furthermore, the order of the items within the hierarchy was randomly selected for each participant at the start of training, remaining unknown to the experimenter. At the end of each block, the overall performance for that block was shown on the screen to keep participants engaged with the task. All subjects underwent a minimum set of three blocks of training. After the third block, and every block thereafter, only performance of the middle pairs, meaning B-C, C-D and D-E, was saved to calculate the exit criteria^11^. If the averaged performance of these pairs for two of the last three blocks was over 66% for one of the hierarchies, the participant stopped receiving feedback for that hierarchy. However, all the premise pairs of this category still appeared on the screen to ensure the same number of trials/appearances for each hierarchy. This continued until the participant reached criteria for all the three hierarchies or a maximum of 10 blocks. In contrast to^10^ and^11^, where the exit criterion was set to 75% accuracy for the middle premise pairs, we used a criterion of 66% to avoid ceiling effects and increase the chances of overnight improvement. On the other hand, we added a more restrictive criterion of 2 blocks out of 3 meeting the threshold, to be sure that the criterion was not achieved by chance. Similarly, to the above-mentioned studies, we only counted the middle premise pairs to evaluate the exit threshold as they are the necessary items for building the inferences.

Session 1 – Immediate test: After criterion was met, participants enjoyed a 5-minute break before proceeding to the immediate test which assessed initial retention of the learned pairs. A similar protocol was used for testing and training with the exception that feedback and sound cues were removed in testing. Subjects were informed that they must select the right item based on previous learning. Participants performed four blocks, with 10 trials per hierarchy. Between blocks, participants solved arithmetic problems to clear short-term memory^51^. Furthermore, pairs from the different hierarchies were randomly interleaved, always with the restriction of not showing the hierarchy explicitly.

Session 2 – Late test: After filling the KSS and SSS questionnaires, participants performed a similar test as before, but this time they were presented with previously learned premise pairs, new inference pairs, and one anchor pair such that a total of 9 pairs were seen instead of just the 5 previously learned. The first new pairs were 3 inference pairs: B > D, B > E and C > E (see Fig. 1d). These pairs are named inference pairs because if you know that B > C and C > D, then you can infer that B > D. The inference pairs can be divided into 1st and 2nd degree of separation. This refers to the number of items between the pair items, for instance between B and D is only one item (C), hence it has first degree separation, as does C-E. On the other hand, there are two items between B and E: C and D. Hence the B-E pair has a second degree of separation and is therefore the most distant pair within a 6-item hierarchy. Additionally, we also added a 4th pair, the anchor pair (A > F) as a control since inference is not needed to obtain this relationship. This is due to the fact that A is always correct and F is always incorrect^51^. Participants were instructed that they might see novel combinations and if that was the case, they should try to make their best guess. At the end of each trial, they rated confidence from −2 (guessing) to +2 (certain) using the up and down arrows. Following a similar protocol to Session 1, participants performed four blocks with math exercises between them.

Session 2 – Sound-Image association test: After a 5-minute break, subjects performed a new sound-image association test with the same structure as the Session 1’s test but without feedback.

Session 3: This used the same tasks in the same order as in Session 2. However, this time participants’ brain activity was not recorded. Finally, the participants performed an Awareness questionnaire (see Supplementary Note 1).

### Closed-loop TMR protocol

The two hierarchies used for TMR and control were counterbalanced across all participants (see SM3). The control hierarchy was not cued during the night. One of the other two hierarchies was assigned to Up state TMR and the other to Down state TMR. Stimulation started after participants entered stable SWS and was halted for arousals or any other sleep stage. Participants were exposed to an extra novel-hierarchy of sounds for each of the two TMR conditions. These novel-hierarchy-sounds, also 200 ms duration, were completely novel to the participants and were included to allow us to distinguish the TMR effect from a normal brain ERP. Each one of the hierarchies was composed of 6 items, each of which were played one by one in the hierarchical order: A, B…, F. The order of both experimental and novel hierarchies and of Up and Down cueing was randomized and counterbalanced across blocks. Each block comprised four hierarchies: experimental Up, novel Up, experimental Down, novel Down (see Fig. 1f). The minimum number of blocks to include a participant in the analysis was 12, meaning 288 cues were presented during the night (12 blocks × 4 hierarchies × 6 items). The mean (SEM) number of cues applied per participant were as follows: 116.05(12.12), 116.52 (12.38), 118.26(12.24) and 118.84 (12.82) for Down Novel, Down Experimental, Up Novel and Up Experimental respectively. Online detection of SOs was based on the detection of the negative half-wave peaks of oscillations. The electrode used as reference for the on-line detection was F3, as frontal regions are predominant SOs areas^52^, band-pass filtered in the slow-wave range (0.5-4 Hz). When the amplitude of the signal passed a threshold of -80uV the auditory stimulus was delivered after a fixed delay of 500 ms^53,54^. Inter-trial intervals were set to a minimum of 4 seconds, that is after every sound played there was a minimum pause of 4 s. The SO detection, auditory stimulation and presentation of the trigger to the EEG recording was via a custom-made Matlab-based toolbox (https://github.com/mnavarretem). Up and Down stimulation was performed in the same manner, with polarity inversion of the signal when necessary.

### EEG recordings

Sleep was recorded using standard polysomnography, including EEG, electromyographic (EMG) and electrooculography (EOG). EEG was recorded using a 64-channel LiveAmp amplifier (Brain Vision^©^). Electrode impedance was kept below 10KΩ and sampling rate was 500 Hz and referenced to Cpz electrode. In addition to the online identification of sleep stages, polysomnographic recordings were scored offline by 3 independent raters according to the AASM criteria^55^, all of them were blind to the periods when the sounds were reactivated.

### EEG analysis

Pre-processing and analysis were all performed with Fieldtrip^56^ and custom Matlab functions. Data were low-pass and high-pass filtered (30 Hz and 0.5 Hz, respectively). Eye and muscle related artefacts were removed using independent component analysis (ICA). Bad channels were interpolated (spline interpolation) and data was re-referenced to linked mastoids. We calculated ERPs by segmenting the cleaned signal into 4 second segments, from −1s before stimulus onset to 3 s afterwards. A final visual inspection of the dataset was performed, and any residual artefact was manually removed. To calculate the differences between stimulation conditions, we averaged across all trials, but for the classification analysis we kept the trial information. To study the time frequency evoked TMR response, we calculated the power spectrum of the signal locked to the TMR cue onset using Morlet wavelets from 4 to 20 Hz with 0.5 Hz resolution and a time window from −1s to 2.4 s in 50 ms steps at the subject level. The width of the wavelet was set to at least 4 cycles per time window, adaptatively to the frequency of interest. Resulting TFRs were then expressed as the relative change of baseline from −1s to 0 ms pre stimulus onset. To assess the accuracy of the closed-loop algorithm, the cleaned data was band-pass filtered (two-pass Butterworth IIR filter) between 0.5 Hz and 2 Hz to assess the slow wave phase. Phase information was obtained through the analytical signal of the filtered data. The angle information was calculated per trial and condition.

### Statistics and reproducibility

Statistical assessment of EEG data was based on nonparametric cluster permutation tests with the following parameters: 2,000 permutations, two-tailed, cluster threshold of *p* < 0.05, and a final threshold of *p* < 0.05 using Fieldtrip toolbox^56^. The time-frequency statistical analysis was restricted to the post-cue interval (0 to 2.4 s) to avoid the natural differences of the Up and Down phases of the SOs before cueing onset. To examine the accuracy of the TMR protocol (circular statistics) the R package Circular was used^57^. Behavioural analysis was performed using robust statistical methods from the R package WRS2^58^ to avoid any possible issues with normality and homoscedasticity assumptions. Repeated measures analysis of variance (RM-ANOVA) or simple 1-way ANOVA analysis was performed accordingly, always keeping the trial information and adding individual differences into the analysis (subjects ID’s). One sample t-test (Students or Wilcoxon signed-rank) tested for difference over chance level (50%) of each group, Condition and Session of interest. Significance of Pearson correlations between classification performance and behaviour used a bootstrap method implemented in R, boot package^59^.

### Classification

Classification of single-trial data was performed using MVPA-light^33^ for each participant and each time point (−1 s to 3 s), hence a 2 ms resolution, using the sleep-ERP values (filtered between 4 and 20 Hz) of the 60 EEG channels as feature. The average number of trials (S.E.M.) per condition were 116 (2.33), 116 (2.26), 118(2.38), 118(2.32) for Down Novel, Down Experimental, Up Novel and Up Experimental conditions. Performance of two classifiers was compared using a linear discriminant analysis (LDA) and a support vector machine with linear kernel (SVM). We used a 5-fold cross-validation method with 2 repetitions and principal component analysis (PCA) to reduce dimensionality (*n* = 20). The data within each fold was z-scored to avoid bias. Additionally, we used two different metrics to evaluate performance of each classifier: traditional accuracy (ACC), defined as % correct predictions, and area under the curve (AUC), or trade-off between the true positive and false positive rates. Once classifiers were calculated for each participant, we performed a between-subject cluster permutation analysis^33^ to determine at what time points the Experimental and Novel sounds were statistically different for each condition.

### Reporting summary

Further information on research design is available in the Nature Portfolio Reporting Summary linked to this article.

### Supplementary information

Supplementary Information

Reporting Summary

## Data Availability

Raw data are available at 10.5281/zenodo.6973571^60^. Data and code to replicate Figs. 2–4 can be found on FigShare: https://figshare.com/projects/Memory_reactivation_in_slow_wave_sleep_enhances_relational_learning_in_humans/189462.

## Code availability

All code for the analysis of this study is available at https://github.com/Contrerana^61^. Code used to replicate Figs. 2–4 can be found on FigShare: https://figshare.com/projects/Memory_reactivation_in_slow_wave_sleep_enhances_relational_learning_in_humans/189462.
