# Supplementary material for: Memory reactivation in slow wave sleep enhances relational learning in humans
Source: Commun Biol. 2024 Mar 8;7:288. doi: 10.1038/s42003-024-05947-7 (PMC10923908; doi:10.1038/s42003-024-05947-7)
Supplement: Supplementary file 1 — Supplementary Information [file 42003_2024_5947_MOESM1_ESM.docx]

**Supplementary materials**

**Supplementary Note 1: Questionnaire**

Page 1:

1. Did you have the impression that some of the pairs of patterns were easier to choose between than others? Yes/No
2. Did you think any of the patterns were ALWAYS correct (no matter what the other pattern was)? Yes/No
3. Did you think that any of the patterns were ALWAYS incorrect (no matter what the other pattern was)? Yes/No
4. Did you have any tricks to memorizing the individual patterns or the pairs of patterns? Yes/No

If yes, explain briefly:

Page 4:

1. Did you think that there was a hierarchy among the patterns seen in training? That is, did you think they could be ranked from "bigger" to "smaller" or from "best" to "worst? Yes/No
2. If you answered Yes, can you write the hierarchy down? (you can use the "names" you have given to the images or any other trick you have used to remember them).

Page 3:

1. In the test phase, did you notice any new combination of patterns taken from those you saw before in the training phase? Yes/No
2. How did you make your choice in the cases? (for example, you guessed, went with instinct). Please explain.

Ten questions were asked at the end of the experiment divided into 4 pages as shown below:

Page 2:

1. Did you give names to the patterns? Yes/No

If yes, can you explain briefly:

1. Did you have the impression that there was some kind of logical rule or order? Yes/No

If yes, can you explain briefly:

**Supplementary Note 2: Sound-Image association**

Participants first learned the sound-image association to 90% criteria to increase chances that the overnight stimulation would trigger the associated image (see Figure 1(A)). They were then tested on these associations but still received feedback to reinforce the learning. In the morning and during session 3 (2-weeks follow-up) they performed the same test but without feedback. A RM-ANOVA was performed to assess any difference in performance across sessions. Accuracy remained almost constantly at ceiling level for the three sessions (M:0.96 SE:0.004, M:0.97 SE:0.004, M:0.96 SE:0.004 respectively) with non-significant differences among them (smallest p=0.55).


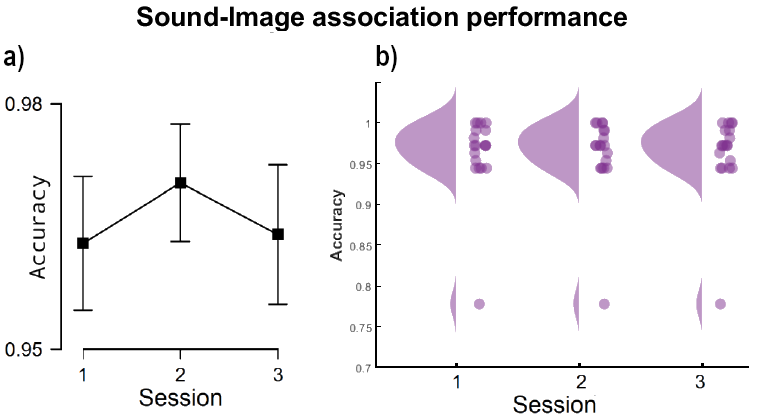


**Supplementary Figure 1:** Sound-Image association performance. Accuracy for each session (evening (1), next morning (2) and 2-weeks follow up (3)) represented in two different ways: a) Horizontal bars represent 95 % confidence intervals. b) Individual points (n=20 for Session 1 and 2, n=17 for Session 3) and their distriibutions. No statistically significant differences were found.

**Supplementary Note 3: Premise Pairs**

Five premise-pairs were presented to the participants in each one of the three testing sessions: A-B, B-C, C-D, D-E, E-F.

**Supplementary Table 1**: Number of times each stimulus (F: Faces, O: objects, S: scenes) is used for each one of the three TMR conditions: Up, Down, Control.

|  | Stimuli |  | Up | Down | Control |  |
| --- | --- | --- | --- | --- | --- | --- |
|  | F |  | 6 | 8 | 6 |  |
|  | O |  | 8 | 5 | 9 |  |
|  | S |  | 8 | 7 | 5 |  |

**Supplementary Table 2:** Premise pair performance for each session (columns) and condition (rows). Accuracy is indicated as: number of trials, mean accuracy and standard error of the mean respectively. (*) Indicates statistically significant difference from chance level (50%).

|  |  | Session 1 |  |  |  | Session 2 |  |  |  | Session 3 |  |
| --- | --- | --- | --- | --- | --- | --- | --- | --- | --- | --- | --- |
|  | n | Mean | SE |  | n | Mean | SE |  | n | Mean | SE |
| Down | 390 | 0.747* | 0.018 | 390 | | 0.747* | 0.019 | 340 | | 0.629* | 0.022 |
| Control | 390 | 0.792* | 0.018 | 390 | | 0.796* | 0.018 | 340 | | 0.696* | 0.021 |
| Up | 390 | 0.796* | 0.018 | 390 | | 0.782* | 0.018 | 340 | | 0.653* | 0.022 |

**Supplementary Note 4: Inference Pairs**

Three inference-pairs were presented to the participants in the last two sessions: B-D, C-E, B-E.

**Supplementary Table 3:** Inference pair performance for Overnight experiment. The first column represents the averaged pair performance, the other two the accuracy divided into the 1^st^ and 2^nd^ degree of separation. Accuracy is depicted for each session and condition of interest and indicated as: number of trials, mean accuracy and standard error of the mean respectively. (*) Indicates statistically significant difference from chance level (50%).

|  |  | Averaged |  |  |  | 1st Degree | |  | |  | 2nd Degree | |
| --- | --- | --- | --- | --- | --- | --- | --- | --- | --- | --- | --- | --- |
|  | n | Mean | SE |  | n | Mean | SE |  | n | | Mean | SE |
| Session 2 |  |  |  |  |  |  |  |  |  | |  |  |
| Down | 234 | 0.464 | 0.028 | 156 | | 0.455 | 0.034 | 78 | 0.481 | | | 0.049 |
| Control | 234 | 0.560* | 0.027 | 156 | | 0.574* | 0.032 | 78 | 0.532 | | | 0.049 |
| Up | 234 | 0.628* | 0.028 | 156 | | 0.587* | 0.035 | 78 | 0.712* | | | 0.045 |
| Session 3 |  |  |  |  |  |  |  |  |  | |  |  |
| Down | 204 | 0.586* | 0.029 | 136 | | 0.577* | 0.036 | 68 | 0.603* | | | 0.050 |
| Control | 2048 | 0.559* | 0.029 | 136 | | 0.555 | 0.036 | 68 | 0.566 | | | 0.050 |
| Up | 204 | 0.583* | 0.031 | 136 | | 0.518 | 0.038 | 68 | 0.713* | | | 0.046 |

**Supplementary Table 4:** RM-ANOVA behavioural results with Session (2 and 3), Condition (Up, Down, Control) and Degree of separation (1^st^ and 2^nd^) as factors. Statistically significant results are highlighted in bold letters.

|  | *F* | *p* | *df1* | *df2* |
| --- | --- | --- | --- | --- |
| Session | 1.969 | 0.170 | 1 | 635.97 |
| **Condition** | 13.97 | **0.002** | 2 | 460.13 |
| **Degree** | 5.804 | **0.017** | 1 | 560.27 |
| **Session*Condition** | 7.868 | **0.021** | 2 | 437.06 |
| **Condition*Degree** | 9.899 | **0.008** | 2 | 460.13 |
| Session*Degree | 0.274 | 0.601 | 1 | 592 |
| Session*Condition*Degree | 0.653 | 0.722 | 2 | 588 |

**Supplementary Table 5:** Post-hoc Session*Condition interaction results. Statistically significant results are highlighted in bold letters.

|  | Ψ | *p* | *Effect size* | *df* |
| --- | --- | --- | --- | --- |
| *Session 2* | | |  |  |
| Control vs Down | 2.25 | 0.214 | 0.218 | 238 |
| Control vs Up | -1.76 | 0.492 | -0.154 | 238 |
| **Down vs Up** | **-4.01** | **<0.001** | -0.372 | 238 |
|  |  |  |  |  |
| *Session 3* | | |  |  |
| Control vs Down | -0.707 | 0.981 | -0.070 | 202 |
| Control vs Up | -0.648 | 0.987 | -0.648 | 202 |
| Down vs Up | 0.059 | 0.997 | 0.059 | 202 |
|  |  |  |  |  |
| Session 2 vs Session 3 | | |  |  |
| Control | -0.105 | 1.0 | 0.04 | 427.00 |
| **Down** | **-3.012** | **0.032** | 0.20 | 430.25 |
| Up | 0.924 | 0.94 | 0.08 | 425.62 |

**Supplementary Table 6:** Post-hoc Degree*Condition interaction results. Statistically significant results are in bold letters.

|  | Ψ | *p* | *Effect size* | *df* |
| --- | --- | --- | --- | --- |
| *Condition only* | | |  |  |
| Control vs Down | 0.826 | 0.687 | 0.059 | 436 |
| **Control vs Up** | **-2.562** | **0.028** | -0.184 | 436 |
| **Down vs Up** | **-3.388** | **0.002** | -0.243 | 436 |
| 1^st^ Degree of separation | | |  |  |
| Control vs Down | 1.43 | 0.709 | 0.117 | 290 |
| Control vs Up | 0.197 | 1.00 | 0.016 | 290 |
| Down vs Up | -1.233 | 0.821 | -0.101 | 290 |
| 2^nd^ Degree of separation |  |  |  |  |
| Control vs Down | 0.00 | 1.00 | <0.001 | 144 |
| **Control vs Up** | **-3.278** | **0.014** | -0.393 | 144 |
| **Down vs Up** | **-3.278** | **0.014** | -0.393 | 144 |
| 1^st^ vs 2^nd^ Degree |  |  |  |  |
| Control | 0.322 | 1.00 | 0.033 | 282.03 |
| Down | -0.086 | 0.959 | -0.085 | 293.48 |
| **Up** | **-3.624** | **0.004** | -0.364 | 323.87 |

**Supplementary Table 7:** Statistical analysis per session for the 2^nd^ Degree of difference per Condition. Statistically significant results are highlighted in bold letters.

| 2^nd^ Degree | *Session 2* | | *Session 3* | |
| --- | --- | --- | --- | --- |
|  | Ψ | *p* | Ψ | *p* |
| Control vs Down | 0.077 | 0.880 | -0.531 | 0.856 |
| **Control vs Up** | **-0.416** | **0.027** | -2.124 | 0.087 |
| **Down vs Up** | **-0.494** | **0.006** | -1.593 | 0.251 |


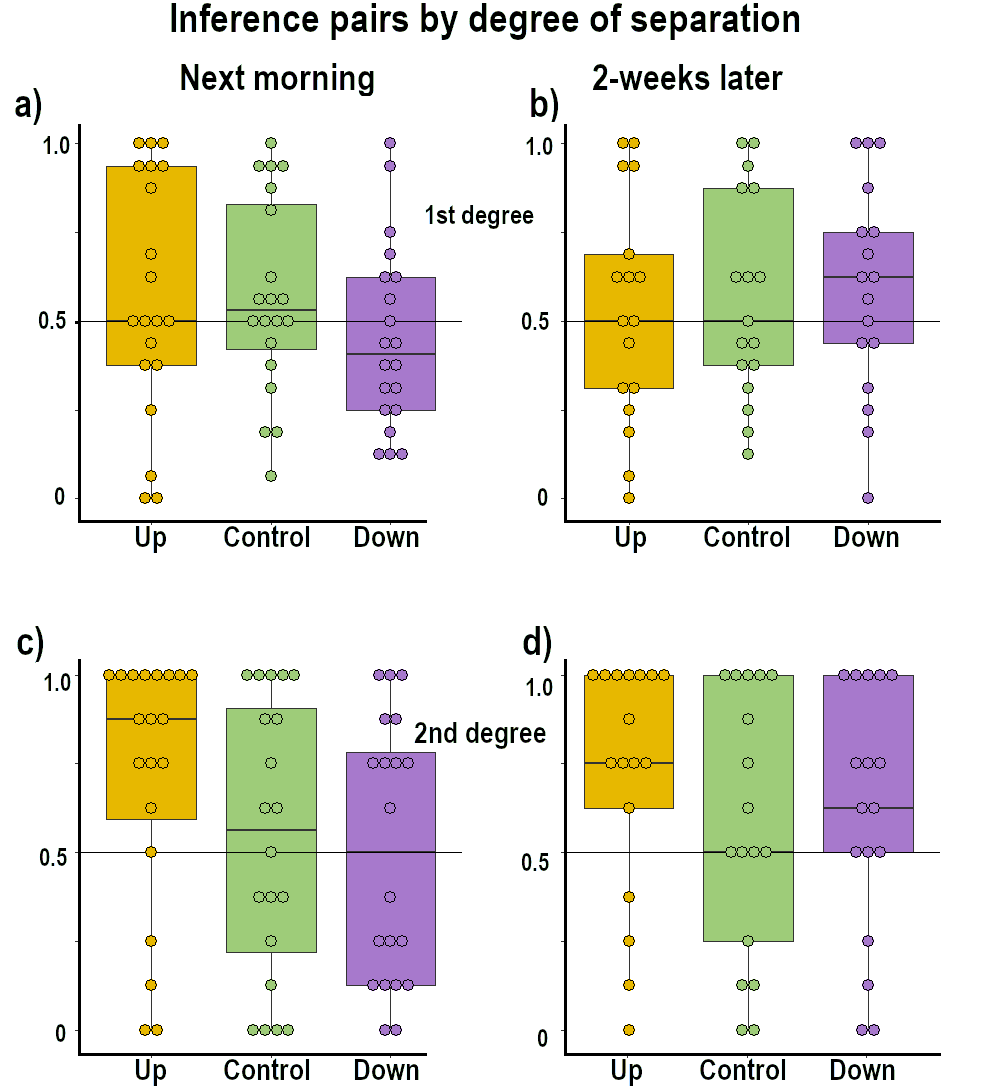


**Supplementary Figure 2:** Inference pairs performance by degree of separation for each condition: Up (yellow), Down (purple) and Control (green). a)1^st^ degree of separation for Session 2 (next morning) and b) Session 3 (2-weeks follow up). c)2^nd^ degree of separation for Session 2 and d) Session 3. Box lines represent the standard notation: middle line is the median and the box extends from the lower quartile (25%) to the upper quartile (75%).

**Time-frequency analysis**

**
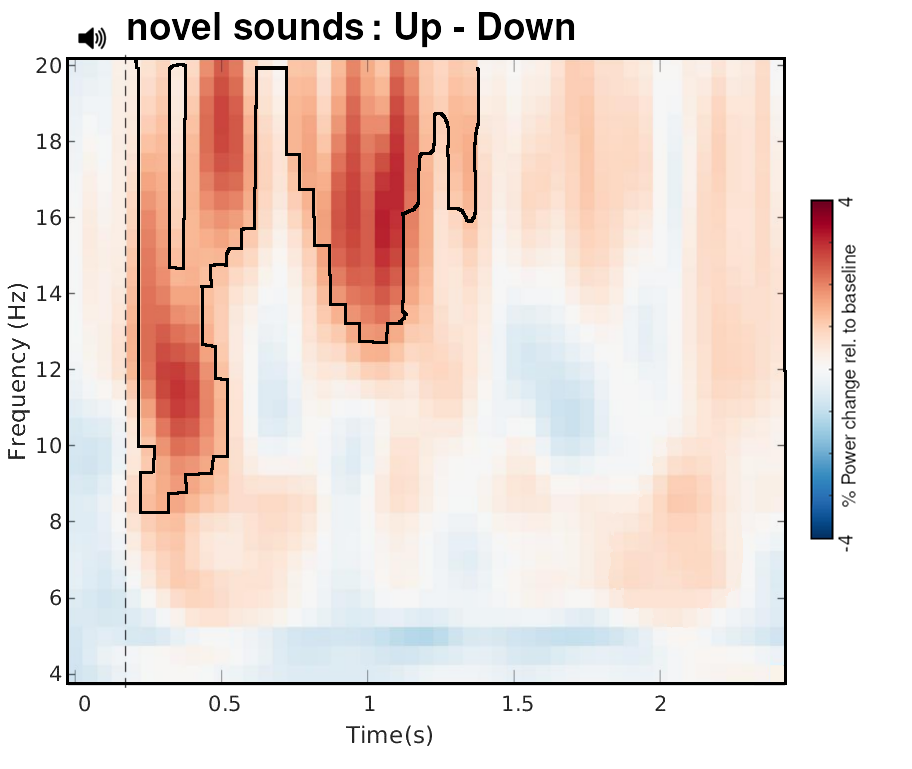
**

**Supplementary Figure 3**: Time-frequency analysis results (grand average at F3 channel) contrasting Up vs Down conditions for Novel sounds. Vertical dashes lines indicate the onset of the auditory TMR cue (200ms). The black contour outlines significant clusters (two tailed, p=0.016).

**Supplementary Note 5: Classification**

We used two classification algorithms, SVM and LDA, with two different performance metrics, AUC (area under the curve) and ACC (accuracy) to distinguish between Experimental and Novel sounds. Cluster statistics resulted in a consistent positive cluster for the Up (see Supplementary Table 8) condition but not significant clusters for the Down condition (see table Supplementary Table 9). Results of the SVM classifier with AUC as performance metrics for both conditions are shown in Supplementary Figure 3. Additionally, we performed correlations between the classification performance and the behavioural results for the Up condition taking both the mean and the peak of the significant classifier cluster. Results for the mean performance within the cluster can be seen in table 4-3 and results for the peak performance within the cluster in Supplementary Table 11.

**Supplementary Table 8:** Significant cluster across different algorithms for Up condition

| Classifier | Performance | Cluster starts (ms) | Cluster ends (ms) | *p* value |
| --- | --- | --- | --- | --- |
| LDA | ACC | 1216 | 1322 | 0.0060 |
| LDA | AUC | 1200 | 1326 | 0.0120 |
| SVM | ACC | 1200 | 1306 | 0.0050 |
| SVM | AUC | 1204 | 1298 | 0.0370 |

**Supplementary Table 9:** Cluster statistic results across different algorithms for Down condition.

| Classifier | Performance | lowest p value |
| --- | --- | --- |
| LDA | ACC | 0.095 |
| LDA | AUC | 0.143 |
| SVM | ACC | 0.106 |
| SVM | AUC | 0.074 |

**Supplementary Table 10:** Correlations results between classifier performance (mean within the significant cluster) and behavioural accuracy of the second degree-inference pairs for the Up condition.

| Classifier | Rho | *p* original | *p* corrected |
| --- | --- | --- | --- |
| LDA ACC | -0.59 | 0.006 | 0.028 |
| LDA AUC | -0.59 | 0.006 | 0.031 |
| SVM ACC | -0.64 | 0.002 | 0.027 |
| SVM AUC | -0.65 | 0.002 | 0.022 |

**
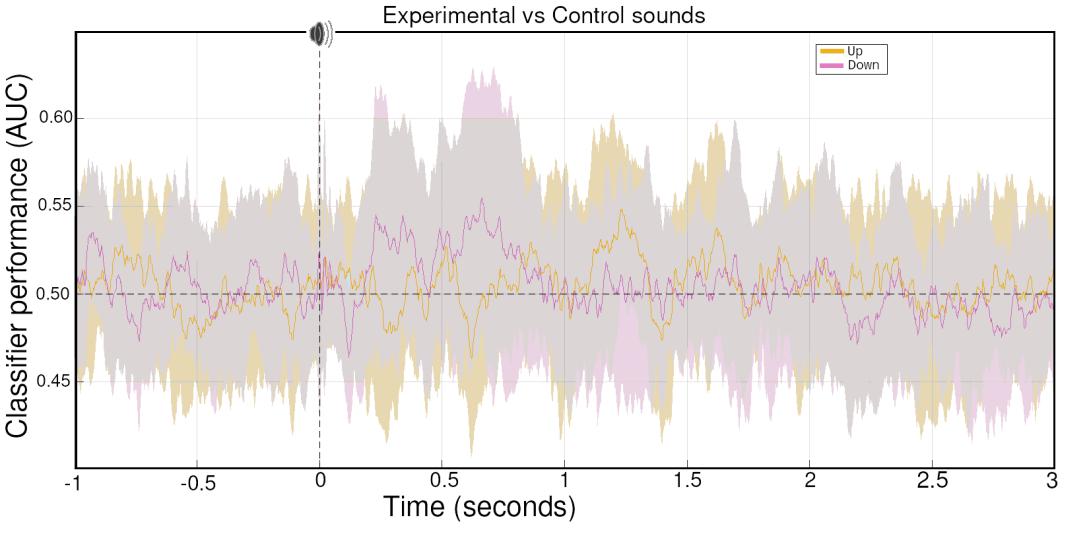
**

**Supplementary Figure 4:** Grand average classifier results for Up (yellowish) and Down (purple) conditions using a SVM with AUC as performance metric. Shadow areas corresponding to the standard deviation across participants.

**Supplementary Table 11:** Correlations results between classifier performance (peak of the significant cluster) and behavioural accuracy of the second degree-inference pairs for the Up condition.

| Classifier | Rho | p original | p corrected |
| --- | --- | --- | --- |
| LDA AUC | -0.49 | 0.039 | 0.063 |
| LDA ACC | -0.48 | 0.039 | 0.065 |
| SVM ACC | -0.57 | 0.012 | 0.042 |
| SVM AUC | -0.56 | 0.012 | 0.047 |
